# Differential activation of inflammatory pathways in A549 type II pneumocytes by *Streptococcus pneumoniae *strains with different adherence properties

**DOI:** 10.1186/1471-2334-6-71

**Published:** 2006-04-11

**Authors:** Rachel L Robson, Natalie A Reed, Rebecca T Horvat

**Affiliations:** 1 Department of Pathology and Laboratory Medicine, University of Kansas Medical Center, 3901 Rainbow Blvd., Kansas City KS 66160, USA

## Abstract

**Background:**

Adherence of *Streptococcus pneumoniae *bacteria to lung cells is a first step in the progression from asymptomatic carriage to pneumonia. Adherence abilities vary widely among *S. pneumoniae *patient isolates. In this study, the binding properties of *S. pneumoniae *isolates and the effects of binding on activation of the Nuclear Factor-Kappa-B (NFκB) pathway and cytokine secretion by type II pneumocytes were measured.

**Methods:**

Mechanisms of high- and low-binding *S. pneumoniae *adherence to A549 cells were investigated by blocking putative receptors on bacteria and host cells with antibody and by eluting choline-binding proteins off of bacterial surfaces. NFκB activation was measured by western blot and immunocytochemistry and cytokine secretion was detected by a protein array.

**Results:**

This study shows that *S. pneumoniae *isolates from pneumonia patients (n = 298) can vary by as much as 1000-fold in their ability to bind to human lung epithelial cells. This difference resulted in differential activation of the NFκB pathway. High-, but not low-binding *S. pneumoniae *used Choline-binding protein A (CbpA) to bind to complement component C3 on epithelial cell surfaces. Interleukin-8 (IL-8) was the only cytokine secreted by cells treated with either low- or high-binding *S. pneumoniae*.

**Conclusion:**

These results indicate that *S. pneumoniae *clinical isolates are not homogeneous in their interaction with host epithelial cells. The differential activation of host cells by high- and low-binding *S. pneumoniae *strains could have implications for the treatment of pneumococcal pneumonia and for vaccine development.

## Background

*Streptococcus pneumoniae *is a leading cause of community-acquired pneumonia [1,2]. Yet *S. pneumoniae *can live harmlessly in the upper respiratory tract of human hosts, and be cultured from the nasopharynx from as many as 40% of the healthy adult population [3,4].

The pathogenicity of *S. pneumoniae *in pneumonia has been the subject of much research [5-9]. Pneumonia caused by *S. pneumoniae *begins when the bacteria move from the upper respiratory tract to the lungs [10]. Although *S. pneumoniae *cannot adhere to healthy ciliated upper respiratory epithelium [11], the bacteria do bind to type II pneumocytes [12,13]. Replication and spread of bacteria through the lung during the first hours of acute *S. pneumoniae *pneumonia is followed by red hepatization, as the lung becomes engorged with blood. Cytokines recruit neutrophils to the site of infection [14]. It is during this stage that the *S. pneumoniae *pneumonia patient is most at risk for severe consequences [15].

The adherence of *S. pneumoniae *to lung epithelial cells is a critical first step in the progression from the asymptomatic carrier state to pneumonia [16]. While studies have indicated that *S. pneumoniae *can use host cell platelet-activating factor receptor (PAFr) as an anchor for adherence [17-19], PAFr is not the sole binding site on human epithelium used by *S. pneumoniae*. *S. pneumoniae *expresses a 75-kDa surface protein, Choline-binding protein A (CbpA) which has been shown to bind to host cells via secreted complement component C3 [20-22]. The binding of C3 on host cells can activate those cells and trigger cytokine release, notably release of the neutrophil-recruiting interleukin-8 (IL-8) [20,23]. Type II pneumocytes have long been known to synthesize and secrete complement component C3 [24], providing a target for *S. pneumoniae *adherence to those cells via CbpA.

It has been noted that 75% of *S. pneumoniae *strains possess genes for CbpA [25] and thus have the potential to bind to C3. This study shows that clinical isolates vary widely in their ability to adhere to lung epithelial cells. A survey of 298 patient isolates from the University of Kansas Medical Center between 1996 and 2001 showed that *S. pneumoniae *strains vary as much as 1000-fold in their ability to bind to a human type II pneumocyte cell line (Robson et al. poster B316, presented at 105^th ^American Society for Microbiology conference, Atlanta, GA, June 2005). Further, high-binding *S. pneumoniae *were selected from a low-binding parent strain, producing two genetically-similar strains that vary in adherence by 2–3 log^10^. The *S. pneumoniae *high binders selected from low-binder parent strains have the same pulsed-field gel electrophoresis patterns and serotypes as their low-binding precursors (Robson et al. poster B75, presented at 102^nd ^American Society for Microbiology conference, Salt Lake City, UT, May 2002)

In this study, the binding properties of naturally-occurring and artificially-evolved high- and low-binding *S. pneumoniae *isolates are compared and the effects of binding on activation of the Nuclear Factor-Kappa-B (NFκB) pathway and Interleukin-8 (IL-8) secretion by type II pneumocytes are measured. Other studies have examined the impact of pneumococcal adherence on the activation of lung epithelial cells; however this is the first to evaluate a substantial number of patient isolates for binding activity, and the first to examine differences between high- and low-binding *S. pneumoniae *in the activation of lung epithelial cells.

## Methods

### Cell culture

A549, a human pneumocyte II cell line, was obtained from ViroMed. Commercially prepared shell vials, containing A549 on glass coverslips, were used for immunocytochemistry. Cells used in other applications were transferred and grown to confluence on sterile culture dishes (Midsci) in commercially-prepared Dulbecco's Modified Eagle's Medium (DMEM) (Sigma) with 10% fetal bovine serum (Atlanta Biologicals) and 1% L-glutamine/penicillin-streptomycin, 1% amphotericin B and 1% MEM essential vitamin mix (Sigma). Cells were transferred to 24-well plates for adherence assays or polystyrene culture dishes for Western blot samples. Cells from passages 4 through 7 were used in all applications.

### Identity of *S. pneumoniae *strains

*S. pneumoniae *isolates were collected from patients at the University of Kansas Medical Center from 1996–2001. All *S. pneumoniae *isolates in this study were from blood or respiratory specimens of hospitalized patients with pneumonia. Medical records were retrospectively reviewed for signs and symptoms of pneumonia. Pneumonia was defined as the presence of a new pulmonary infiltrate by chest radiograph, confirmed by a radiologist as compatible with pneumonia.

Additionally the patient must have had at least two signs or symptoms consistent with a lower respiratory tract infection: temperature of 38°C or greater, new onset or increased cough, purulent sputum, crackles, or dyspnea. Isolates from patients with other diagnoses were not included in this study. The Human Subjects Committee at the University of Kansas Medical Center approved the study protocol and the information was recorded in a manner that preserved patient confidentiality. These procedures are in accordance with the guidelines for human experimentation of the U.S. Department of Health and Human Services.

Naturally-occurring high- and low-binders were chosen from these patient isolates for further experiments. Evolved high-binding *S. pneumoniae *were descendants of low-binding strains: two patient isolates, serotypes 3 and 18C, and ATCC 6301, serotype 1. The reference strain ATCC 6301 was obtained from the American Type Culture Collection.

All *S. pneumoniae *used except for ATCC 6301 had different genetic backgrounds, as determined by pulsed-field gel electrophoresis using established techniques [26]. Artificially-evolved high-binding strains had the same PFGE pattern and serotype as their respective low-binding parent strains.

### Adherence assay and selection for adherence

24-well plates containing 200,000 A549 cells per well were washed three times with Muller-Hinton broth (MHB) (Remel) at room temperature. After washing, each well was inoculated with 10^7 ^bacteria in 1 mL additive-free DMEM, and incubated at 37°C, 5% CO_2 _for two hours. The cells were then washed three times with room temperature MHB, and cells were lysed with 0.02% trypsin (Sigma) for 30 minutes. Bacterial viability was not affected by 0.02% trypsin. The A549 cell lysate was then diluted and plated on sheep blood agar (SBA) (Remel), using a standard spread plate technique. Colony counts from the cell lysate dilutions determined the number of adherent bacteria per 100 A549 cells.

The evolved high binding strains of *S. pneumoniae *were derived from low binding parent strains by using sequential passage on fresh A549 monolayers. When selecting for adherence, the adherent bacteria from the previous passage on A549 cells were used to inoculate new cell layers of A549 for subsequent passages.

In choline-elution experiments, bacteria were soaked in sterile 2% choline (Sigma) in saline for 20 minutes, centrifuged and resuspended in sterile saline before adding the bacteria to A549 cells in 24-well plates.

In all adherence experiments, A549 viability immediately prior to the final treatment with 0.02% trypsin was over 90%, as determined by trypan blue exclusion.

### Blocking of receptors on *S. pneumoniae *and A549

The CbpA proteins on *S. pneumoniae *were blocked by soaking bacteria in sterile saline supplemented with either1:100 rabbit anti-CbpA, or 1:100 normal rabbit serum as the negative control, for 30 minutes at room temperature with gentle shaking. The bacteria were then washed in sterile saline prior to being added to A549 cells to assess adherence.

To block the C3 and PAF-receptor on the A549 cells, the epithelial cells were treated with 1:100 rabbit anti-human C3 antibody, 1:100 goat anti-human PAFr antibody (Santa Cruz Biotechnology), or 1:100 normal rabbit serum as the negative control for 2 hours. The cells were then washed with MHB and inoculated with a standard suspension of bacteria.

### Serotyping

Serotyping was performed using bead agglutination, as previously described [27].

### Western blot for IκB

Protein preparations from A549 cells treated with high- and low-binding bacterial strains (MOI = 50 bacteria/cell) were quantified using a protein quantification kit (BioRad). 50 μg of protein was electrophoresed in a 30% polyacrylamide gel, transferred to a polyvinylidine fluoride (PVDF) membrane (BioRad), Ponceau stained, blocked in 5% nonfat dry milk (NFDM) in Tris-buffered saline-Tween (TBST), and probed with rabbit anti-human IκB (Santa Cruz Biotechnology) in 5% NFDM-TBST, with horseradish-peroxidase (HRP) conjugated donkey anti-rabbit IgG (Santa Cruz Biotechnology) used as a secondary antibody. IκB bands were visualized using ECL substrate (Amersham Biosciences) and autoradiography.

Computerized densitometry was used to analyze changes in IκB band density relative to negative controls. Membranes were stripped and reprobed with mouse anti-human actin antibody to confirm that equivalent amounts of total protein had been loaded into each well.

### Western blot for eluted CbpA

Choline-binding proteins were eluted from *S. pneumoniae *as described above. Supernatants were collected and sterile-filtered and proteins were precipitated using a standard tricholoracetic acid technique. The proteins were electrophoresed on a 30% polyacrylamide gel and transferred to PVDF as described above. Membranes were Ponceau stained, blocked in 5% NFDM-TBST, and probed with 1:100 rabbit anti-CbpA (kindly provided by Drs. Elaine Tuomanen and Margaret Hostetter). HRP-conjugated donkey anti-rabbit IgG was used as a secondary antibody.

### Immunocytochemistry

A549 cells grown on glass coverslips in shell vials (ViroMed) were used for immunocytochemistry. Cell layers were washed with MHB and inoculated with 10^7 ^bacteria per vial, as in adherence assays. At the end of the incubation period (4, 6, or 8 hours at 37°C, 5% CO_2_), cell layers were washed three times with ice-cold phosphate-buffered saline (PBS) and fixed overnight at 4°C in 5% formalin in PBS. Immunocytochemistry was conducted using a kit (BioGenex) with 1:25 rabbit anti-NFκB (Santa Cruz Biotechnology) used as a primary antibody. Slides were examined at 400 x.

### Cytokine array

Supernatants from A549 cells treated with high and low binding *S. pneumoniae *isolates were assayed for the presence of 42 human cytokines using a RayBio^® ^Human Cytokine Antibody Array III, following manufacturer's instructions (Raybiotech). Cytokine array luminesence was visualized with ECL substrate and autoradiography.

### Measurement of IL-8 concentrations

IL-8 was measured from the supernatant of treated epithelial cells using the Bio-plex suspension array system with fluorescently dyed Luminex microspheres (BioRad). Culture supernatant was collected from cells at 30 minutes, 1 hour, 2 hours, 4 hours, 6 hours and 8 hours after inoculation with bacteria. The culture supernatant was stored at -70°C until tested. Assays were performed in a 96-well filtration plate (Millipore) with 5,000 beads coated with human IL-8 antibody. The standards, samples or blanks were mixed with beads in a final volume of 100 μl and incubated for 30 minutes at 25°C with continuous shaking. After three washes, biotinylated antibodies were added for 30 minutes. Beads were again washed three times and streptavidin-phycoerythrin was added for 10 minutes. The fluorescence intensity of the beads was measured using the Bio-Plex array reader. Bio-Plex Manager software was used for data analysis. The level of IL-8 detection was 7.8 pg/ml. IL-8 standards were assayed in triplicate with each assay; n = 7 samples run per treatment per timepoint.

### Statistical analysis

All statistical analysis was done in Statistical Analysis Software (SAS). Graphs were made using Microsoft Excel.

### Pulsed-field gel electrophoresis

Genomic DNA from artificially-evolved and naturally occurring *S. pneumoniae *strains was prepared as previously described [26]. The fragments were resolved using a GenePath System (BioRad) set on the "Enterococcus" running program.

## Results

### *S. pneumoniae *clinical isolates vary in ability to bind to epithelial cells

Clinical isolates of *S. pneumoniae *(n = 298) were assayed for ability to bind to A549 cells, a human type II pneumocyte cell line. Bacterial isolates varied by more than 1000-fold in their ability to adhere to cells. Table 1 summarizes *S. pneumoniae *clinical isolates' adherence abilities into 4 distinct categories: low, low-moderate, moderate and high. The majority (66%) of *S. pneumoniae *assayed were low binders, adhering to lung cells at an average rate of 0.82 ± 0.52 bacteria per 100 A549 cells. A small minority (2%) of *S. pneumoniae *were high binders, adhering to lung cells with an average rate of 2,360 ± 770 bacteria bound per 100 cells. The remainder of the isolates were low-moderate or moderate binders. Differences in adherence ability among the four categories was statistically significant (one-way ANOVA p-value << 0.005; F = 167.84 where F_critical _= 2.74) and reproducible. Neither serotype nor binding ability predicted for invasive disease, defined here as the isolate having been cultured from blood (Cochran-Mantel-Haenszel test, p = 0.41 and p = 0.82, respectively). No relationships were observed between serotype and binding ability, or between antibiotic resistance and binding ability of *S. pneumoniae*.

**Table 1:** Observed adherence properties of naturally-occurring *S. pneumoniae *clinical isolates

**Binding category**	**% of total clinical isolates observed (n = 298)**	**Average *S. pneumoniae *bound per 100 A549 cells ± standard deviation**^a^
low	66% (n = 194)	0.82 ± 0.52
low-moderate	19% (n = 56)	5.2 ± 1.8
moderate	14% (n = 42)	30.7 ± 11.2
high	2% (n = 6)	2360 ± 770

^a ^MOI = 50 bacteria/cell

### High-binding *S. pneumoniae *isolates use CbpA to bind to C3 on epithelial cell surfaces

To investigate pneumocyte receptors used by high- and low-binding *S. pneumoniae*, PAFr and C3 on the A549 cells were blocked with specific antibodies. Pre-treating A549 cells with anti-PAFr antibody did not reduce adherence of high-binding *S. pneumoniae *strains (data not shown). However, adherence of low-binding *S. pneumoniae *strains to A549 cells was slightly reduced (40%) by blocking PAFr on host cells with antibody (data not shown).

**Figure 1:**
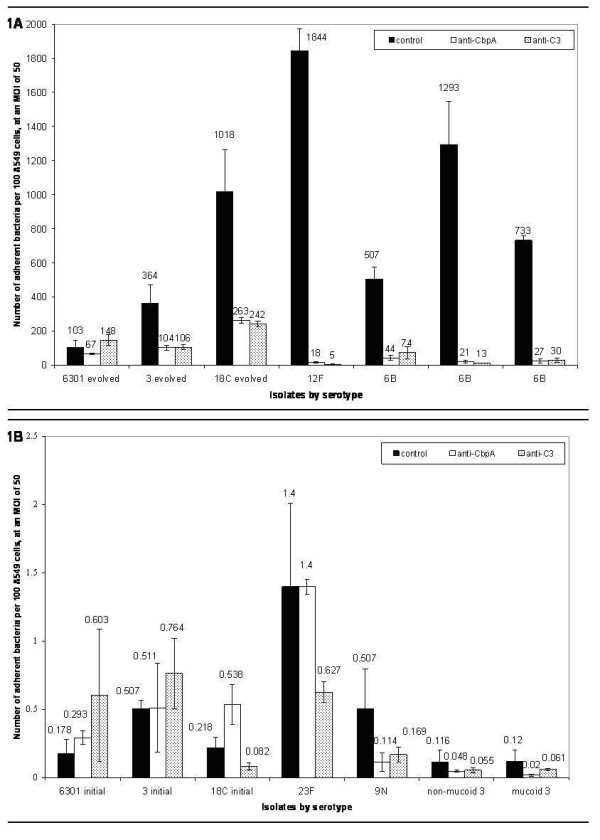
Adherence of high-binders, but not low-binders, is reduced by blocking CbpA or C3. Solid bars, negative control; open bars, bacteria pre-treated with anti-CbpA antibody; dotted bars, A549 cells pre-treated with anti-C3 antibody. Negative control A549 cells pre-treated with normal rabbit serum. All values are mean ± one standard deviation, based on three independent experiments. Mean values are above each bar. The Y-axis units differ on graphs 1A and 1B. **1A. **High-binding *S. pneumoniae *are identified by serotype along X-axis. 6301 evolved, 3 evolved, and 18C evolved are high-binding descendants of low-binding parent strains. Isolates identifed as 6 B all share that serotype but have different PFGE patterns. **1B. **Low-binding *S. pneumoniae *strains are identified by serotype along X-axis. 6301 parent, 3 parent, and 18C parent are initial low-binding strains of 6301 evolved, 3 evolved, and 18C evolved.

Blocking cell surface-bound complement component C3 with anti-human C3 antibody significantly reduced adherence of all but one high binding *S. pneumoniae *strain (Figure 1A). Alternatively, only one low-binding *S. pneumoniae *strain bound less avidly to A549 cells treated with anti-C3 antibody than to control cells (Figure 1B).

Since choline-binding proteins (Cbps) on bacteria have been implicated in *S. pneumoniae *adherence to host cells, their involvement in the differential adherence of *S. pneumoniae *strains was investigated. All Cbps were eluted off of the surfaces of high- and low-binding *S. pneumoniae *using standard methodology [28]. Elution of choline-binding proteins from *S. pneumoniae *surfaces eliminated bacterial adherence to A549 cells, among both high-binding and low-binding strains. Thus, choline-binding proteins are necessary for adherence of both high- and low-binding *S. pneumoniae *to epithelial cell surfaces.

To determine whether CbpA is responsible for *S. pneumoniae *adherence to A549 cells, bacteria were treated with anti-CbpA (provided by Drs. Hostetter and Tuomanen). Similar to blocking host cell C3 with anti-C3 antibody, blocking pneumococcal CbpA with anti-CbpA antibody significantly reduced adherence of all but one high-binding *S. pneumoniae *strain to A549 cells (Figure 1A). Blocking CbpA on low-binding *S. pneumoniae *with antibody did not significantly reduce adherence to A549 cells (Figure 1B).

The removal of CbpA from bacterial surfaces by elution in 2% choline solution was confirmed by Western blot. Membranes were probed with anti-CbpA. Eluents from all high-binding and all but one low-binding *S. pneumoniae *strains exhibited CbpA-positive bands at 75 kDa. These data indicate that all but one isolate used in these experiments express CbpA, and that this protein was successfully removed from bacterial surfaces by choline elution (data not shown).

### High-binding *S. pneumoniae *activate NFκB pathway

**Figure 2:**
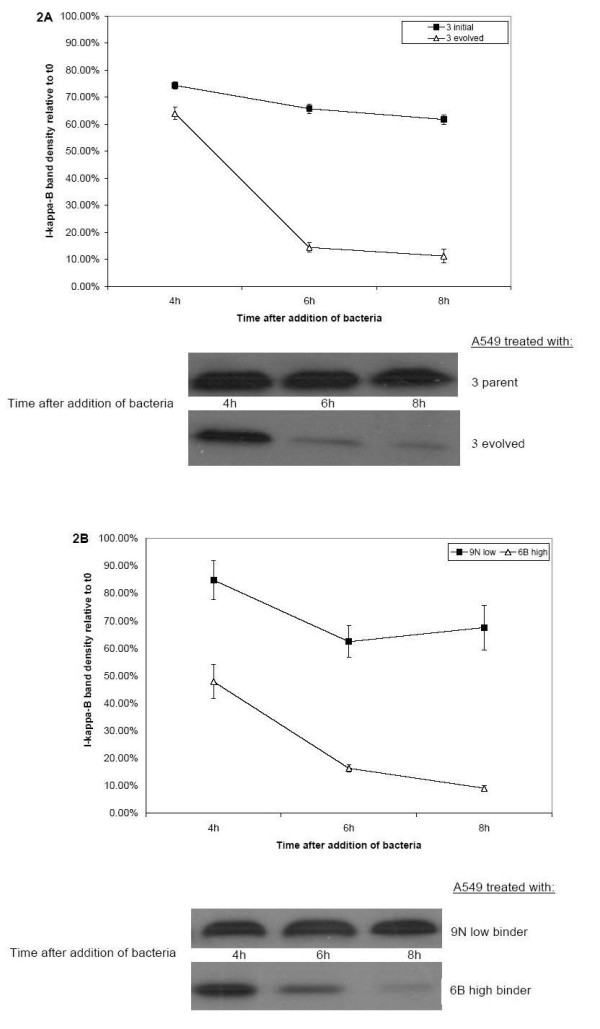
High-binders induce prolonged degradation of IκB compared to low-binders. All values are mean ± one standard deviation, based on three independent experiments. **2A. **IκB degradation induced by serotype 3 evolved high-binder and 3 low-binding parent strains. Closed squares, IκB level relative to time zero in A549 treated with low-binder. Open triangles, IκB level relative to time zero in A549 treated with high-binder. Representative Western blots for IκB are shown below each line graph. **2B. **IκB degradation induced by serotype 9N low-binding clinical isolate and serotype 6B high-binding clinical isolate. Closed squares, IκB level relative to time zero in A549 treated with low-binder. Open triangles, IκB level relative to time zero in A549 treated with high-binder. Representative Western blots for IκB shown below line graph.

Activation of the NFκB pathway in A549 was detected by the degradation of the NFκB inhibitor, IκB. Figure 2 shows Western blots for IκB from A549 cells treated with high- and low-binding *S. pneumoniae *strains at different time points. Both high- and low-binding *S. pneumoniae *induced IκB degradation in A549 cells. However, at the 4, 6 and 8 hour time points, IκB levels were significantly lower among all cells treated with high-binding *S. pneumoniae *compared to all cells treated with low-binding strains (two-way ANOVA comparison of IκB band densities for cells treated with high and low binders at 4, 6, and 8 hours relative to zero timepoint). Table 2 summarizes statistical analysis of IκB degradation in A549 after exposure to high-and low-binding *S. pneumoniae *strains. Figure 2A shows IκB loss induced by an artifically-evolved high-binder and its genetically-similar low-binding parent strain. Figure 2B shows IκB loss induced by two unrelated high- and low-binder clinical strains. Binding of C3 on A549 surfaces by anti-C3 antibody did not induce IκB degradation.

**Table 2:** Percentage of IκB remaining in A549 after addition of high- and low-binding *S. pneumoniae*^a^

	**Time after addition of bacteria**			
	
Treatment of A549 cells^b^	0 hours	4 hours	6 hours	8 hours
low binders	100%	81.3%	65.6%	62.1%
high binders	100%	60.8%	34.9%	25.1%

ANOVA p-value^c^	0.423	2.3 × 10^-7^	1.3 × 10^-7^	1.1 × 10^-8^

^a ^Combined average of IκB levels from cells treated with 7 low-binders or 7 high-binders. Each *S. pneumoniae *isolate was tested in triplicate thus each measurement is the average from 21 different experiments at each time point.

^b ^A549 cells were incubated with an MOI of 50 bacteria/cell for the listed time periods. Proteins from the cell culture were then harvested for the analysis of IκB degradation.

^c ^Two-way analysis of variance comparing IκB degradation in A549 treated with low binders and high binders for each time period. IκB degradation measured as described in materials and methods.

**Figure 3:**
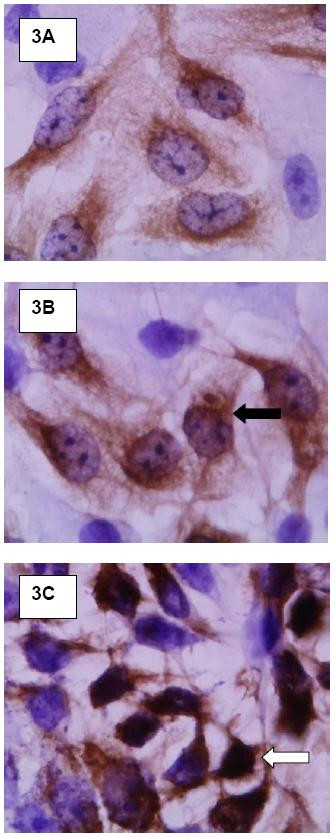
NFκB migrates to the nucleus after cell interaction with high-binders, but not low-binders, 4 hours after inoculation with bacteria. NFκB is detected as a dark brown stain against a pale lavender background. 3A, negative control. 3B, A549 incubated with serotype 3 low-binders (Black arrow shows NFκB protein in the cytoplasm). 3C, A549 incubated with serotype 3 high-binders (White arrow shows NFκB protein detected in the nucleus).

Immunocytochemistry confirmed the activation of NFκB pathway by demonstrating the migration of NFκB from the cytoplasm into the nucleus in A549 cells treated with high-binding, but not low binding, strains (Figure 3).

### Cytokine activation by high and low binding *S. pneumoniae*

**Figure 4:**
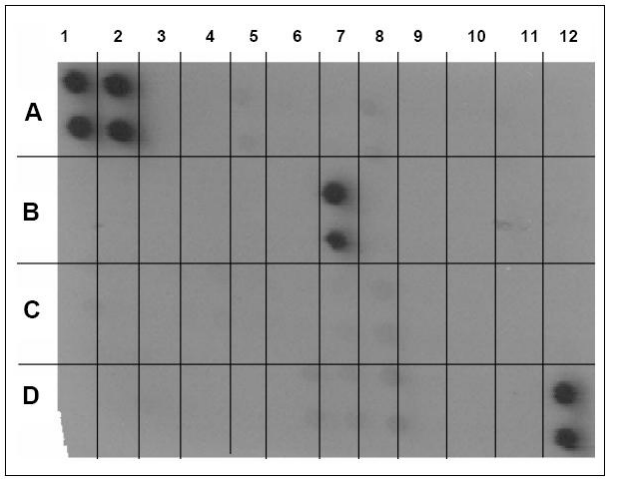
Supernatants from A549 cells treated with high- and low-binding *S. pneumoniae *were evaluated with a RayBio^® ^Human Cytokine Antibody Array III, which detects 42 different human cytokines. 1A and 2A, positive control; 3A and 4A, negative control; 5A, ENA-78; 6A, GCSF; 7A, GM-CSF, 8A, GRO; 9A, Gro-α; 10A, I-309; 11A, IL-1α; 12A, IL-1β; 1B, IL-2; 2B, IL-3; 3B, IL-4; 4B, IL-5; 5B, IL-6; 6B, IL-7; 7B, IL-8; 8B, IL-10; 9B, IL-12; 10B, IL-13; 11B, IL-15; 12B, IFN-γ; 1C, MCP-1; 2C, MCP-2; 3C, MCP-3; 4C, MCSF; 5C, MDC; 6C, MIG; 7C, MIP-1; 8C, RANTES; 9C, SCF; 10C, SDF-1; 11C, TARC; 12C, TGF-β; 1D, TNF-α; 2D, TNF-β; 3D, EGF; 4D, IGF-1; 5D, angiogenin; 6D, oncostatin M; 7D, thrombopoietin; 8D, VEGF; 9D, PDGF; 10D, leptin; 11D, negative control; 12D, positive control. The positive result for IL-8, in position 7B, with negative results for all other cytokines, was observed for A549 cells treated with both high- and low-binding bacteria. Experiment replicated with four different high-binding, and four different low-binding, *S. pneumoniae *strains.

Cytokine release was evaluated using a protein array that included 42 cytokines. Culture fluid from A549 cells inoculated with high- and low-binding *S. pneumoniae *was tested after 2 hours of incubation. The only cytokine detected after exposure to either high- or low-binding strains was IL-8 (Figure 4). To determine whether high- or low-binding *S. pneumoniae *induced different amounts of IL-8 secretion from A549 cells, a Luminex bead system was used. No significant difference in IL-8 concentrations was observed in the supernatants of cells treated with high- versus low-binders at any time point prior to 8 hours. Cells treated with low-binders released significantly more IL-8 at 8 hours than did those treated with high-binders (Wilcoxon Rank-Sum, two-tailed p-value < 0.05). Negative controls induced only negligible IL-8 secretion, near the detection limit of the system (1.6 pg/mL). Measurement of other cytokines, TNF-α and IL-4, was below the detection limit of the system at all time points for all cells.

## Discussion

Adherence to host cells is the first step in the progression from *S. pneumoniae *carriage to disease [3]. While adherence to host cells by *S. pneumoniae *has been shown in a number of studies [10,12,17,20-22,29,30], this is the first investigation to compare adherence across a large sample of *S. pneumoniae *isolates from pneumonia patients. An important conclusion of this study is that *S. pneumoniae *isolates' ability to adhere to lung cells vary by more than 1,000-fold. Additionally, *S. pneumoniae *with higher binding activity to host cells have a prolonged activation of the NFκB pathway.

Patients infected with high-binding isolates could be at increased risk compared to patients infected with the more common low-binding isolates. While high-binding *S. pneumoniae *strains are relatively uncommon among patient isolates, they could still be important clinically. The United States' Centers for Disease Control expects at least 100,000 cases of pneumococcal pneumonia every year in the United States alone [31]. Thus, 2,000 of those patients would be expected to be infected with high-binding strains if the percentages reported here are representative of *S. pneumoniae *isolates overall. Although this investigation does not compare outcomes of patients infected with high- and low-binding *S. pneumoniae*, studies are in progress to evaluate this association. These studies require the evaluation of more patients since high-binding bacteria comprise such a small minority (2%) of patient isolates.

There was no correlation between isolate ability to bind to host cells and serotype, although other studies have shown a relationship between pathogenicity and serotype [32] and invasiveness and serotype [33]. In this study, 18 serotypes were found among 298 patient isolates. While 3 of 6 high-binding isolates were serotype 6B, this capsular type was not statistically linked to increased adherence, since 6B is a common serotype in this sample (n = 39; 13%).

High-binding *S. pneumoniae *used a bacterial surface protein, CbpA, to bind to C3 on lung epithelial cells, while low-binding strains did not use CbpA or C3. This interaction between CbpA and C3 has been noted by others [21,22]. CbpA is also capable of binding to other cellular receptors, such as the polymericimmuno globulin receptor (pIgR) on nasopharyngeal epithelial cells [30,34] and PAFr [17,29]. In this study blocking host PAFr did not reduce the adherence of high-binders to A549 cells.

Orihuela et al. observed that CbpA is required for nasopharyngeal colonization, progression from the nasopharynx to the lower respiratory tract, and the progression from bacteremia to meningitis in a mouse model but is not needed for *S. pneumoniae *survival in murine lungs or blood [35]. In contrast, Paton and Berry showed that CbpA does not contribute to *S. pneumoniae *pathogenicity in mice infected via intraperitoneal injection [36]. Future studies are needed to elucidate the importance of CbpA on virulence in humans infected with *S. pneumoniae*.

Others have shown that high-binding *S. pneumoniae *strains have a transparent colony morphology, while colonies of low-binding *S. pneumoniae *are likely to be opaque [12,37] Further, it has been observed that transparent *S. pneumoniae *strains contain more CbpA than do opaque strains [21] and that individual *S. pneumoniae *isolates can fluctuate between highly-adherent transparent and less-adherent opaque phases [37,38]. Phase variation was not noted when comparing high-binding *S. pneumoniae *isolates with low-binding *S. pneumoniae *isolates. All clinical strains used exhibited stable binding activity and colony morphology over at least 4 years and after numerous passages from the freezer to agar. Additionally, artificially-evolved high binders had the same colony morphology as did their low-binding progenitors. Thus, in this instance it does not seem that colony morphology was responsible for differences in binding ability of these clinical isolates. It is possible that certain colony morphotypes were selected out in the host or during the infectious process. Those studies would require additional patient isolates and extensive review of patient history. Interestingly, four of the high-binding *S. pneumoniae *isolates came from patients under the age of 5 years. This observation could imply that high-binding *S. pneumoniae *using CbpA are more prevalent in children than in adults. This interesting finding requires further study.

CbpA has been considered as a vaccine candidate. CbpA is immunogenic in rabbits and *S. pneumoniae *human volunteers [39,40]. Recently, Zhang et al. found that children with higher serum levels of anti-CbpA antibodies had a reduced risk of *S. pneumoniae *carriage [41]. Thus, CbpA-mediated binding to host cells could be an important contributor to *S. pneumoniae *pathogenicity and could have implications for the development of vaccines directed against CbpA.

In this study, high-binding *S. pneumoniae *were observed to strongly activate the NFκB pathway in lung epithelial cells. NFκB activation was independent of serotype and genetic background. Although NFκB activation often leads to secretion of many different cytokines, in this study only IL-8 was released by cells treated with either high- or low-binding *S. pneumoniae*. Epithelial cells surprisingly produced more IL-8 after 8 hours of incubation with low binding *S. pneumoniae *than with high binding isolates. This indicates differences in intracellular pathways within the epithelial cells after activation of different receptors by *S. pneumoniae*. Additionally, the interaction and recruitment of other cell types to the site of infection may be effected by this differential activation. A study by Jones et al. showed that IL-1 and TNF-α were rapidly released by murine lungs in response to *S. pneumoniae *infection [42]. Thus, although the results from the current study indicate a difference in the initial interaction of *S. pneumoniae *with host epithelial cells alone, further investigation in the presence of other cell types is needed to understand the implications of this difference in epithelial cell activation.

NFκB activation and IL-8 secretion by pneumocytes recruit neutrophils to the lung [43,44]. While neutrophils kill bacteria, they also damage lung tissue [44]. It is not clear whether the increased IL-8 secretion in pneumocytes treated with low-binding *S. pneumoniae *is ultimately protective or destructive. It is also unclear why the increased NFκB activation seen in cells treated with high-binding *S. pneumoniae *did not result in increased IL-8 secretion.

## Conclusion

This study has shown that *S. pneumoniae *isolates have differences in ability to bind to human lung epithelial cells. This difference was reproducible and resulted in differential activation of the NFκB pathway and IL-8 secretion. High binding *S. pneumoniae *adhered via an interaction between CbpA and C3. These results indicate that *S. pneumoniae *are not homogeneous in their interaction with host epithelial cells and may have differences in pathogenicity. Further studies are needed to elucidate the pathogenic implication of this difference in binding and activation of epithelial cells.

## Competing interests

The author(s) declare that they have no competing interests.

## Authors' contributions

RR and RH conceived of the study. RR conducted all evolution of adherence experiments, pulsed-field gel electrophoresis, and all immunoblotting and immunocytochemistry. RR and NR conducted all serotyping and adherence assays. NR designed and compiled the patient database and performed statistical analyses. RH collected cytokine data.

## Pre-publication history

The pre-publication history for this paper can be accessed here:


